# Effects of artesunate on the malignant biological behaviors of non-small cell lung cancer in human and its mechanism

**DOI:** 10.1080/21655979.2022.2042141

**Published:** 2022-03-31

**Authors:** Peng Hu, Chengyao Ni, Pen Teng

**Affiliations:** Department of Cardiac and Great Vessel Surgery, First Affiliated Hospital, College of Medicine, Zhejiang University, Hangzhou, Zhejiang Province, China

**Keywords:** Apoptosis, artesunate, invasion, lung cancer, migration

## Abstract

We aimed to assess the effects of artesunate (ART) on the proliferation, migration, invasion and apoptosis of the non-small cell lung cancer cells A549 and H1299. The effects of ART and carboplatin (CBP) alone or in combination on the viability of A549 and H1299 cells were evaluated by MTT assay. The effects of 30 μg/ml ART on cell invasion, migration and apoptosis were evaluated by Transwell assay, scratch assay and flow cytometry, respectively. The protein expressions of human antigen R (HuR) and MMP-9 after treatment with 30 μg/ml ART for 48 h were detected by Western blotting. After 48 h of treatment, 9 μg/ml ART in combination with 7 μg/ml CBP exerted a mild synergistic effect on cell viability. The migration rates of cells treated with 30 μg/ml ART and number of invasive cells were significantly lower, and the apoptosis rates were higher than those of the DMSO-treated group. HuR and MMP-9 expressions in cells treated with 30 μg/ml ART for 48 h were significantly lower than those of the DMSO-treated group. ART suppresses the proliferation, migration and invasion of A549 and H1299 cells and induces their apoptosis, probably being associated with decreased expressions of HuR and MMP-9 proteins.

## Graphical Abstract

**Figure:**
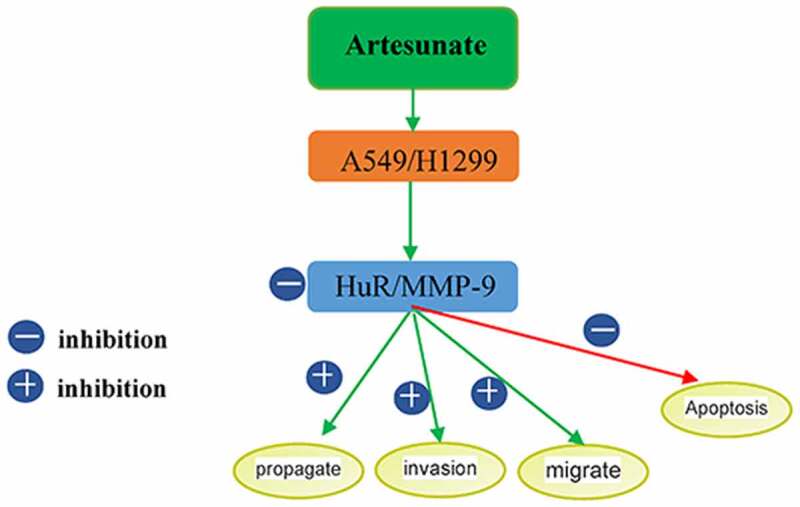

## Introduction

Lung cancer is the leading cause for cancer deaths worldwide. Among them, non-small cell lung cancer (NSCLC) accounts for over 80% of all lung cancer cases, which easily undergoes invasion and metastasis [1]. At present, chemotherapy still plays an important role in the treatment of malignant tumors. For example, platinum-based doublet chemotherapy remains valuable for the postoperative adjuvant therapy of NSCLC. However, conventional cytotoxic drugs greatly damage normal cells and easily lead to acquired drug resistance, accompanied by unsatisfactory clinical treatment outcomes [2,3]. Although molecular targeted agents have worked well for the treatment of NSCLC in recent years, it is urgent to develop a new generation of chemotherapeutic drugs.

**Table 1.** Effects of ART on viability of A549 and H1299 cells

Group	Dose	A549		H1299	
		Inhibition rate (%)	IC50 (μg/ml)	Inhibition rate (%)	IC50 (μg/ml)
DMSO-treated group	0	**/**		**/**	
ART (μg/ml)	10	24.89	28.8	26.37	27.2
	20	43.27		44.38	
	40	56.17		57.19	
	80	78.26		76.28	
	120	89.46		87.43

**Table 2.** Effects of CBP on viability of A549 and H1299 cells

Group	Dose	A549		H1299	
		Inhibition rate (%)	IC50 (μg/ml)	Inhibition rate (%)	IC50 (μg/ml)
DMSO-treated group	0	**/**		**/**	
CBP (μg/ml)	5	12.67	23.5	12.78	22.9
	10	33.25		34.31	
	20	42.12		43.11	
	40	77.21		78.89	
	80	97.57		99.56	

The process of invasion mainly includes adhesion of tumor cells to the extracellular matrix (ECM), degradation of ECM components and subsequent penetration of tumor cells into adjacent tissues. Matrix metalloproteinases (MMPs) have been reported to participate in the degradation of ECM. MMP-9 can cleave type IV collagen, the main component of the basement membrane, so it plays an important role in tumor invasion [4]. It has previously been reported that artesunate (ART) can regulate the level of MMP-9 [5].

Currently, cancers are still mainly treated by platinum-based or combined regimens. However, long-term high-dose use easily causes serious adverse reactions, including nephrotoxicity, gastrointestinal reactions, ototoxicity and hematological toxicity. Therefore, platinum-based drugs should be combined with other drugs with different mechanisms of action to enhance the antitumor effects and to mitigate adverse reactions and the incidence rate of resistance [6].

Artemisinin-based drugs have played a unique role in the treatment of malaria. Besides, the antitumor effects of artemisinin-based drugs have attracted widespread attention [7–10]. These drugs evidently affect the expressions of metastasis-related factors in tumor cells [11]. Human antigen R (HuR), as an RNA-binding protein of the embryonic lethal abnormal vision family, can regulate the stability of various mRNAs [12]. HuR can escape nuclease degradation through binding of the RNA recognition motif to AU-rich elements (ARE) in the 3’ untranslated region of mRNA. It is involved in the regulation of post-transcriptional expression of a variety of metastasis-related molecules and highly expressed in multiple human tumor cells [13,14]. Considering that many tumor-related molecules downregulated by artemisinincontaining cis-acting element ARE, the target gene of HuR, we herein intended to study whether ART affected the expression or biological function of HuR.

Motivated by this, we assessed the effects of ART on the proliferation, migration, invasion, apoptosis and expressions of HuR and MMP-9 proteins of human lung cancer cell lines A549 and H1299, aiming to provide experimental evidence for elucidating the mechanism by which ART-based drugs combat lung cancer metastasis.

## Methods

### Cell lines and main reagents

Human lung cancer cell lines A549 and H1299 were purchased from Cell Bank, Shanghai Institutes for Biological Sciences (China). ART was bought from Guilin South Pharmaceutical Co., Ltd. (China). Carboplatin (CBP) was purchased from Sigma-Aldrich (USA). MTT assay kit was obtained from Thermo Fisher Scientific (USA). Matrigel was provided by BD (USA). Transwell was purchased from Corning (USA). Annexin V-FITC kit was bought from Shenzhen Neobioscience Technology Co., Ltd. (China). Rabbit anti-polyclonal antibodies against HuR, MMP-9 and β-actin were obtained from Abcam (USA). HRP-labeled secondary antibody was provided by Beijing Zhongshan Golden Bridge Biotechnology Co., Ltd. (China).

### Cell culture

A549 and H1299 cells were pipetted into 15 ml centrifuge tubes and 5 ml of complete medium was added to it, and the tubes were centrifuged for 5 min to discard the supernatant. After the addition of 5 ml of complete medium, the cells were mixed evenly and cultured in a constant-temperature incubator.

### Detection of lung cancer cell proliferation by MTT assay

A549 and H1299 cells in the logarithmic phase were digested by 0.25% trypsin solution. Then the cells were inoculated into 96 well plates at the concentrations of 5 × 10^4^/ml and 20 × 10^4^/ml, respectively, incubated overnight in a 37°C incubator with 5% CO_2_ and treated with drug-containing culture media. Three replicate wells were set for each group. After 48 h of culture, 20 μl of MTT solution (5 mg/ml) was added into each well, followed by 4 h of incubation. After the supernatant was discarded, 150 μl of DMSO was added into each well and shaken at room temperature for 10 min to dissolve violet particles. Then the optical density (OD) of each well at 490 nm was measured with a SPECTRAmax M5 microplate reader. The viability of the cells was calculated as follows. Viability (%) = (OD_experimental well_ – OD_blank well_)/(OD_control well_ – OD_blank well_) × 100%. Finally, IC50 values were calculated by GraphPad Prism 5.0 software [15]. DMSO-treated cells were used as a negative control.

### Detection of lung cancer cell migration by scratch assay

A549 and H1299 cells were cultured in 6-well plates at the concentration of 5 × 10^5^/well. When they grew to cover the entire well, a sterile 10 μl pipette was used to scratch evenly at the bottom of each well. Floating cells were washed with preheated PBS and the culture medium was added with or without 30 μg/ml ART, and the cell scratches were photographed. After 6-well plates were cultured in the constant-temperature incubator for 16 h, the cell scratches were photographed again. The cell migration distances in the presence or absence of ART treatment were compared. Migration rate (%) = (distance measured immediately after scratching – distance measured 16 h after scratching)/ distance measured immediately after scratching × 100% [16].

### Detection of lung cancer cell invasion by Transwell assay

Matrigel (50 μg/ml) was added into Transwell chambers with 8.0 μm polycarbonate membranes. Subsequently, the chambers were placed in a 37°C incubator for about 5 h to allow Matrigel to solidify. A549 and H1299 cells were digested, collected by centrifugtion at 12,000 × g for 5 min and adjusted to a concentration of 5 × 10^5^/ml. Then 100 μl of cell suspension was added into each chamber. After the addition of the culture medium with or without 30 μg/ml ART, the culture plate was placed in a 37°C incubator for 48 h. At the end of culture, the media in the upper and lower chambers were sucked out, and 250 μl of 0.1% crystal violet solution was thereafter added to stain the cells for 10–15 min. Matrigel in the upper chamber was gently removed with a cotton swab. Five visual fields were selected to observe each well under a microscope (magnification: 400×), and invasive cells were counted [17].

### Detection of lung cancer cell apoptosis by flow cytometry

A549 and H1299 cells were treated with 0 or 30 μg/ml ART for 48 h. The cells were collected by centrifugation at 12,000 × g for 5 min and counted. The cells were washed with precooled PBS, centrifuged twice, then resuspended by using an appropriate amount of binding buffer and adjusted to the concentrations of 2–5 × 10^5^/ml. Afterward, 5 μl of annexin V-FITC was added into the cell suspension and mixed gently. Three minutes later, 10 μl of 20 μg/ml propidium iodide solution was added to incubate the cells in the dark for 10 min at room temperature. Finally, an appropriate amount of binding buffer was added for flow cytometry [18].

### Measurement of HuR and MMP-9 protein expressions by western blotting

A549 and H1299 cells treated with 30 μg/ml ART or not for 48 h were collected by centrifugation at 12,000 × g for 5 min. Total cell protein was extracted, and protein concentration was determined by the BCA method. Subsequently, 20 μg protein sample was resolved by 12% SDS-PAGE, and the product was transferred onto a PVDF membrane. The membrane was blocked with 5% skimmed milk at room temperature for 3 h and incubated overnight with anti-HuR and anti-MMP-9 antibodies (1:1,000 diluted) at 4°C using β-actin as the internal reference, followed by incubation with corresponding second antibodies (1:20,000 diluted) at room temperature for 2 h. TBST was used to wash the membrane three times, 10 min each time, and fresh luminescent solution was prepared. The membrane was incubated in the luminescent solution for 3 min, exposed to X-ray film, developed and imaged in the dark. The images of the bands were scanned, and the gray values of HuR and MMP-9 bands were measured by Image-Pro Plus software to calculate relative protein expressions. The experiment was repeated three times [19].

### Statistical analysis

All data were statistically analyzed by SPSS19.0 software. The quantitative data were expressed as mean ± standard deviation. Intergroup comparisons were performed by the *t*-test, and multigroup comparisons were conducted with a one-way analysis of variance. P < 0.05 or 0.01 was considered statistically significant.

## Results

### Effects of ART and CBP alone on viability of A549 and H1299 cells

Halatsch et al. found that ART augmented the antiproliferative effects of temozolomide on glioblastoma U87MG and A172 cells [20]. Additionally, ART has well-documented therapeutic effects on esophageal cancer cells (Eca109/ADM) and liver cancer HepG2 cells [21].

MTT assay showed that ART and CBP alone had obvious inhibitory effects on cell viability (Tables 1 and 2). The IC50 values of ART and CBP for A549 cells were calculated to be 28.8 μg/ml and 23.5 μg/ml, respectively, and those for H1299 cells were 27.2 μg/ml and 22.9 μg/ml, respectively.

### Effects of ART and CBP in combination on viability of A549 and H1299 cells

Low-dose ART (6 μg/ml, 9 μg/ml, 12 μg/ml and 15 μg/ml) and different concentrations of CBP with similar inhibition rates (5 μg/ml, 7 μg/ml, 10 μg/ml and 12 μg/ml) were combined to treat A549 and H1299 cells. After 48 h of treatment, 9 μg/ml ART in combination with 7 μg/ml CBP began to exert a mild synergistic effect on cell viability (Table 3).

**Table 3.** Effects of ART and CBP in combination on viability of A549 and H1299 cells

Group		A549		H1299	
	Dose (μg/ml)	Inhibition rate (%)	CI	Inhibition rate (%)	CI
DMSO-treated group	0	**/**		**/**	
ART	6	14.37		15.28	
	9	21.38		22.76	
	12	26.28		27.43	
	15	28.11		29.08	
CBP	5	7.89		8.04	
	7	18.25		19.27	
	9	28.79		29.43	
	12	35.78		36.26	
ART+CBP	6 + 5	28.47	1.02	29.02	1.03
	9 + 7	36.78	0.92	37.26	0.91
	12 + 9	47.28	0.79	48.92	0.77
	15 + 12	61.24	0.66	62.35	0.65

### ART inhibited the migration of A549 and H1299 cells

**Figure 1.**
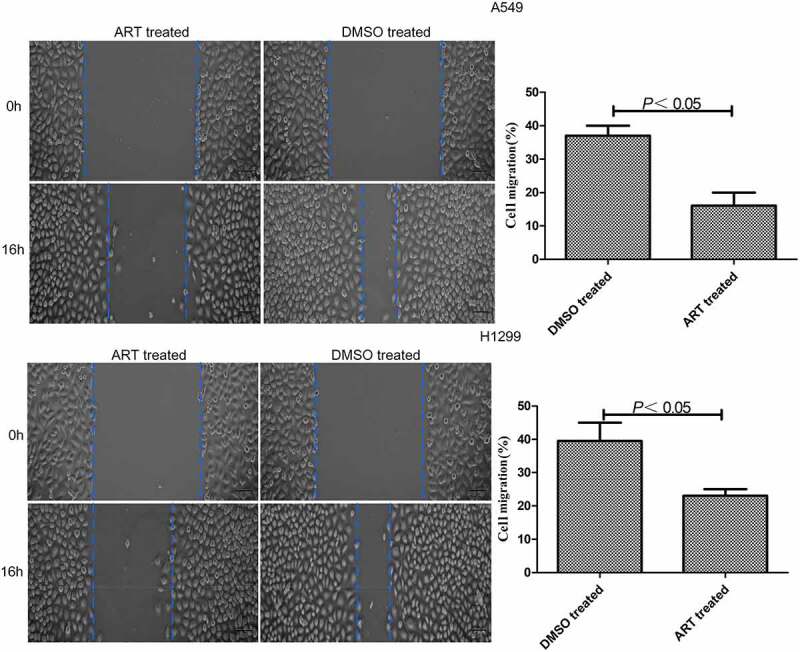
Inhibitory effects of ART on migration of A549 and H1299 cells evaluated by scratch assay. The experiments were performed in triplicate.

The migration rates of A549 and H1299 cells treated with 30 μg/ml ART were significantly lower than that of the DMSO-treated group (Figure 1). Therefore, 30 μg/ml ART markedly suppressed the migration of these cells.

### ART inhibited invasion of A549 and H1299 cells

**Figure 2.**
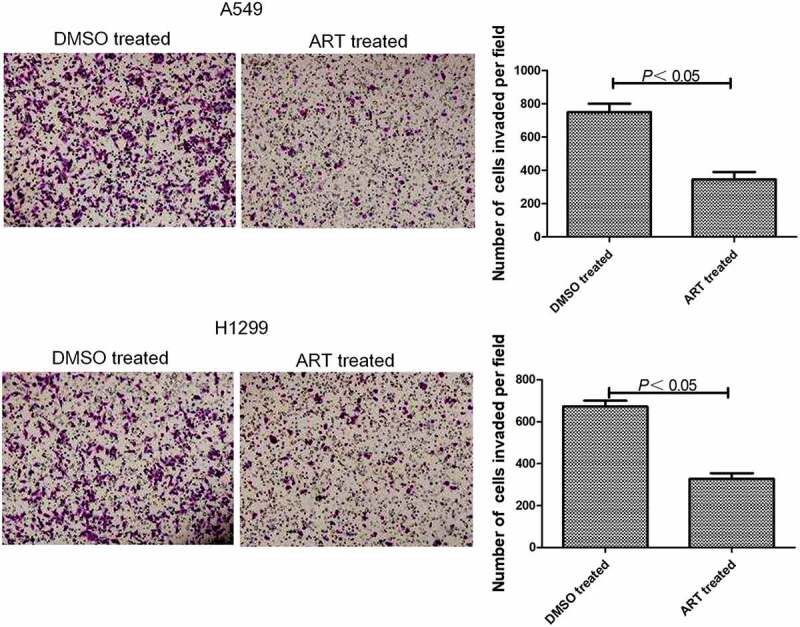
Inhibitory effects of ART on invasion of A549 and H1299 cells assessed by Transwell assay. The experiments were performed in triplicate. Magnification: 400 ×.

Transwell assay showed that 30 μg/ml ART significantly reduced the number of A549 and H1299 cells penetrating Matrigel into the lower chamber (Figure 2). Thus, 30 μg/ml ART obviously suppressed the invasion of these cells.

### ART promoted apoptosis of A549 and H1299 cells

**Figure 3.**
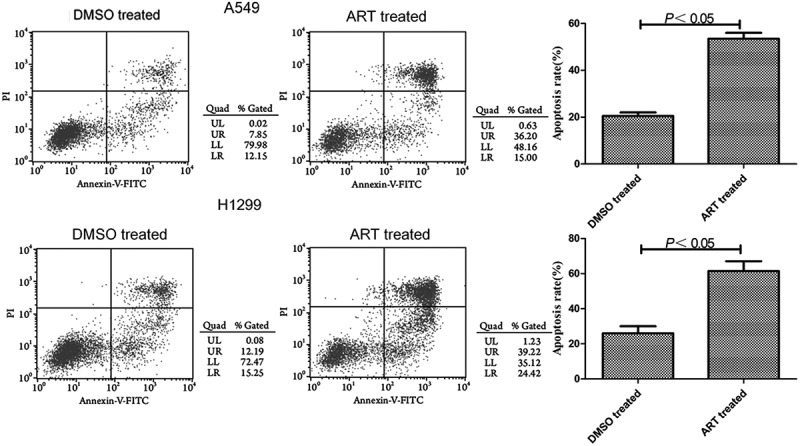
Promotive effects of ART on apoptosis of A549 and H1299 cells evaluated by flow cytometry. The experiments were performed in triplicate.

The apoptosis rates of A549 and H1299 cells treated with 30 μg/ml ART were significantly higher than that of the DMSO-treated group (Figure 3). Accordingly, 30 μg/ml ART apparently facilitated the apoptosis of these cells.

### ART decreased HuR and MMP-9 protein expressions in A549 and H1299 cells

**Figure 4.**
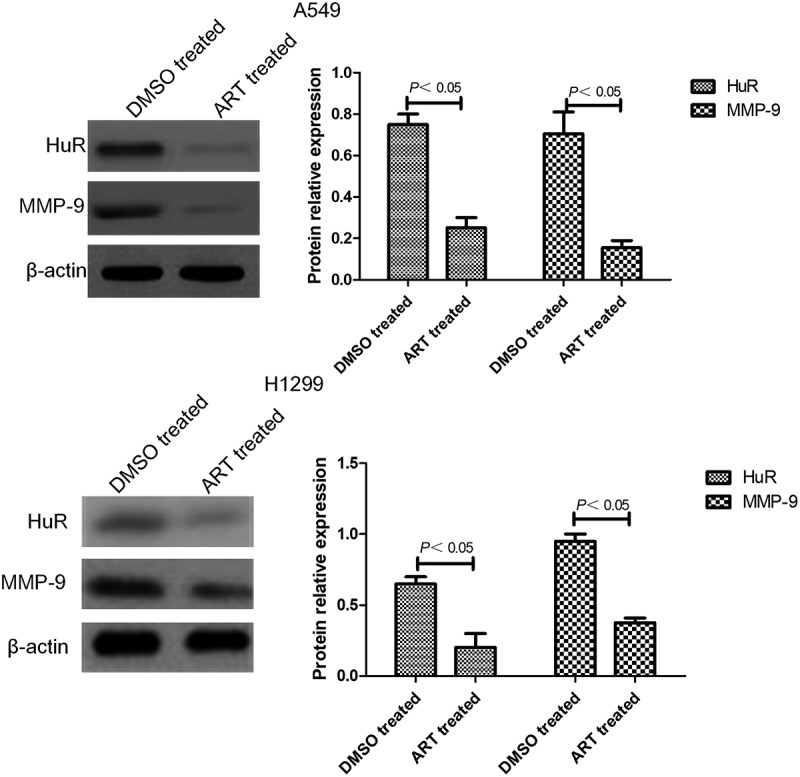
Western blotting results of HuR and MMP-9 protein expressions in A549 and H1299 cells treated with ART. The experiments were performed in triplicate.

Western blotting showed that the expression levels of HuR and MMP-9 in A549 and H1299 cells treated with 30 μg/ml ART for 48 h were significantly lower than those of the DMSO-treated group (Figure 4).

## Discussion

Lung cancer is the most common cause for cancer death around the world, and it is mostly prone to local spread and distant metastasis upon diagnosis. The recurrence and metastasis rates remain high even if radical surgery is performed for early-stage patients, and the 5-year survival rate is lower than 10% [22]. Currently, chemotherapy is still the major method for treating lung cancer, while traditional cytotoxic drugs cause great damage to normal cells, thus leading to drug resistance easily [23].

In recent years, it is well known that the active ingredients extracted from Chinese herbal drugs have antitumor effects. ART is one of the derivatives of artemisinin, an antimalarial drug with complete intellectual property rights developed by Chinese scientists, which has excellent water solubility and antimalarial effects. In addition, artemisinin and its derivatives also have antitumor, anti-inflammatory and antifibrotic effects [24–26]. Currently, the potent antitumor activities of artemisinin and its derivatives have been highlighted. According to the study of the American Cancer Society [27], ART is cytotoxic against 55 kinds of malignant tumor cells, including colon cancer, leukemia, melanoma and breast cancer cells. ART can cause cell cycle arrest and apoptosis in triple-negative MDA-MB-468 and HER2-enriched SK-BR-3 breast cancer cells [28]. It has also been confirmed that ART can inhibit the proliferation and induce the apoptosis of lung cancer cells cultured *in vitro* dose-dependently [29]. In this study, ART was able to significantly inhibit the proliferation of A549 and H1299 cells in dose-dependent manners. ART (30 μg/ml) suppressed the migration and invasion of A549 and H1299 cellsH1299 cells, and induces and also promoted their apoptosis. The above findings further suggest that ART may be a potential anti-lung cancer drug. In the meantime, we herein found that 9 μg/ml ART combined with 7 μg/ml CBP exerted a mild synergistic effect, being in accordance with a previous literature [30].

Tumor invasion and metastasis are the major causes for the death of patients. The recurrence and metastasis rates of NSCLC are among the top in those of primary malignant tumors. Therefore, it is necessary to clarify the mechanism of invasion and metastasis of lung cancer at the molecular level and to explore suitable preventive strategies. Tumor invasion and metastasis is composed of adhesion, protein degradation and migration at the molecular level. First, tumor cells adhere to ECM, vascular endothelium and target cells. Then hydrolase is secreted to degrade and to destroy the ECM and capillary basement membrane. Afterward, tumor cells invade the peripheral tissues or enter the blood circulation or lymphatic system, ultimately triggering metastasis. Currently, the effects of ART on the invasion and metastasis of malignant tumor cells have rarely been reported. It has been reported that ART can regulate the expression of proteolytic enzymes and affect the synthesis and degradation of ECM [31,32]. Rasheed et al. found for the first time that ART suppressed lung cancer invasion and metastasis *in vivo* by targeting extracellular proteases [33]. MMP-9 is an essential cytokine for the invasion and metastasis of tumor cells and also the largest-molecular-weight enzyme in MMPs, which can degrade ECM and the major structural protein (collagen IV) of the basement membrane. MMP-9 is highly expressed in a variety of highly invasive malignant tumors. According to clinical studies, MMP-9 has high expression not only in lung cancer tissues but also in serum of patients, as an important reference index for the early diagnosis and prognostic evaluation of NSCLC patients [34,35]. Inhibiting the expression of MMP-9 in lung cancer H1299 cells cultured *in vitro* can suppress early metastasis. As an RNA-binding protein, HuR can regulate the stability of mRNA [36,37], while MMP-9 is a downstream molecule of HuR. HuR is closely associated with the clinical stage and lymph node metastasis of NSCLC as an independent factor for the poor prognosis of patients [14]. In this study, ART suppressed the protein expressions of HuR and MMP-9, indicating that it may inhibit the proliferation, migration and invasion but promote the apoptosis of human lung cancer A549 cells by regulating the expressions of HuR and MMP-9.

Regardless, this study still has some limitations. We only assessed the effects of ART on the protein expression levels of HuR and MMP-9. In the future, we will use pathway inhibitors to further explore the action mechanism of ART.

## Conclusion

In conclusion, ART obviously suppressed the proliferation, migration and invasion but promoted the apoptosis of human lung cancer A549 and H1299 cells and also reduced the protein expression levels of HuR and MMP-9. Such effects may be related to a decline in the expression levels of HuR and MMP-9. The results provide references for in-depth research on the antitumor effects of ART and the biological behaviors of HuR in tumors.
